# Supplementary material for: Quantitative somatosensory assessments in patients with persistent pain following groin hernia repair: A systematic review with a meta-analytical approach
Source: PLoS One. 2024 Jan 31;19(1):e0292800. doi: 10.1371/journal.pone.0292800 (PMC10830060; doi:10.1371/journal.pone.0292800)
Supplement: S1 File — (DOCX) [file pone.0292800.s002.docx]

**S2 File. Search String**

Search string:

*’Chronic Pain’[MeSH Terms] OR ’pain, postoperative’[MeSH Terms] OR chronic pain*[Text Word] OR postoperative pain*[Text Word] OR persistent pain*[Text Word] OR postsurgical pain*[Text Word]’)* ***AND***

*(’Hernia, Inguinal’[Mesh] OR ’Herniorrhaphy’[Mesh] OR groin hernia repair*[Text Word] OR inguinal hernia repair*[Text Word] OR inguinal herniorrhaphy*[Text Word] OR groin hernia herniorrhaphy*[Text Word] OR inguinal hernia*[Text Word]’)* ***AND***

*(’Sensory Thresholds’[Mesh] OR ’Pain Threshold’[Mesh] OR ’Pain Measurement’[Mesh] OR quantitative sensory test*[Text Word] OR QST[Text Word] OR somatosensory test*[Text Word] OR sensory profile*[Text Word] OR sensory threshold*[Text Word] OR pain threshold*[Text Word] OR warmth detection test*[Text Word] OR cold detection test*[Text Word] OR heat pain threshold*[Text Word] OR cold pain threshold*[Text Word] OR pressure pain threshold*[Text Word] OR quantitative sensory test*[Text Word]’)*
