# Supplementary material for: Quantitative somatosensory assessments in patients with persistent pain following groin hernia repair: A systematic review with a meta-analytical approach
Source: PLoS One. 2024 Jan 31;19(1):e0292800. doi: 10.1371/journal.pone.0292800 (PMC10830060; doi:10.1371/journal.pone.0292800)
Supplement: S1 Table — (DOCX) [file pone.0292800.s003.docx]

**S3 Table. Table 4 – NOS (Cross-sectional studies)**

Summary of the quality assessments of cross-sectional studies using the Newcastle-Ottawa Scale (NOS). As the NOS is not inherently designed for cross-sectional studies, a modified version of the scale was used for the quality assessments [1, 2].

| Assessed by: | Refs. | Author | Year | Title | Selection Bias Assessment  (Maximum 4 stars) | | | | Comparability  (Max 2 stars) | Outcome (Maximum 3 stars) | | Total score  (Maximum  9 stars) |
| --- | --- | --- | --- | --- | --- | --- | --- | --- | --- | --- | --- | --- |
|  |  |  |  |  | Representativeness  of the sample | Sample size | Non-respondents | Ascertainment of the exposure (risk factor) | Confounding factors are controlled | Assessment of the outcome | Statistical Test |  |
| AD/EKJ/MW | 18 | Mikkelsen | 2004 |  | * | NO | NO | ** | ** | * | * | 7 |
| AD/EKJ/MW | 26 | Aasvang | 2009 | Preoperative pain and sensory function in groin hernia | * | NO | * | ** | N/A | * | * | 6 |
| EKJ/AD/MW | 27 | Aasvang | 2010 | Persistent sensory dysfunction in pain-free herniotomy | * | * | NO | ** | ** | * | * | 8 |
| AD/EKJ/MW | 37 | Kristensen | 2012 | Chronic pain after inguinal hernia repair in children | * | NO | NO | ** | N/A | * | NO | 4 |
| AD/EKJ/MW | 35 | Ergönenç | 2017 | Persistent postherniorrhaphy pain following inguinal hernia repair | * | NO | NO | ** | ** | * | * | 7 |

1. Herzog R, Álvarez-Pasquin MJ, Díaz C, Del Barrio JL, Estrada JM, Gil Á. Are healthcare workers’ intentions to vaccinate related to their knowledge, beliefs and attitudes? a systematic review. BMC Public Health. 2013;13(1):154.

2. Wells GA, Wells G, Shea B, Shea B, O'Connell D, Peterson J, et al., editors. The Newcastle-Ottawa Scale (NOS) for Assessing the Quality of Nonrandomised Studies in Meta-Analyses. https://www.ohri.ca/programs/clinical_epidemiology/oxford.Asp (accessed 01/03/2023)
