# Supplementary material for: Quantitative somatosensory assessments in patients with persistent pain following groin hernia repair: A systematic review with a meta-analytical approach
Source: PLoS One. 2024 Jan 31;19(1):e0292800. doi: 10.1371/journal.pone.0292800 (PMC10830060; doi:10.1371/journal.pone.0292800)
Supplement: S2 Table — (DOCX) [file pone.0292800.s004.docx]

**S4 Table. Table 5 – NOS (Cohort studies)**

Summary of the quality assessments of cohort studies using the Newcastle-Ottawa Scale (NOS) [1]

| Assessed by: | Refs. | Author | Year | Title | Selection Bias Assessment (Maximum 4 stars) | | | | Comparability  (Max 2 stars) | Outcome (Maximum 3 stars) | | | Total score (Max 9) |
| --- | --- | --- | --- | --- | --- | --- | --- | --- | --- | --- | --- | --- | --- |
|  |  |  |  |  | Representativeness of the exposed cohort | Selection of the non-exposed cohort | Ascertainment of exposure | Demonstration  that outcome of  interest was not present at start of study | Comparability of cohorts based on the design or analysis | Assessment of the outcome | Was follow-up long enough for outcomes to occur | Adequacy of follow up of cohorts |  |
| AD/EKJ/MW | 12 | Aasvang | 2007 | Ejaculatory pain: a specific postherniotomy pain syndrome? | * | * | * | NO | * | Self report | * | NO | 6 |
| AD/EKJ/MW | 24 | Aasvang | 2008 | Neurophysiological characterization of postherniotomy pain | * | * | * | NO | * | Self report | * | * | 7 |
| AD/EKJ/MW | 34 | Beldi | 2008 | Postoperative hypoesthesia and pain: | * | * | * | NO | * | Self report | * | * | 7 |
| AD/EKJ/MW | 15 | Aasvang | 2009 | The effect of mesh removal | * | * | * | NO | ** | NO | * | * | 7 |
| AD/EKJ/MW | 36 | Kalliomäki | 2009 | Persistent pain after groin hernia surgery: a qualitative analysis of pain | * | * | * | NO | ** | NO | * | * | 7 |
| AD/EKJ/MW | 33 | Aasvang | 2010 | Predictive risk factors for ersistent postherniotomy pain | * | * | * | * | ** | NO | * | * | 8 |
| AD/EKJ/MW | 25 | Aasvang | 2010 | Heterogeneous sensory processing in persistent postherniotomy pain | * | * | * | NO | * | NO | * | * | 6 |
| AD/EKJ/MW | 38 | Linderoth | 2011 | Neurophysiological characterization of persistent pain after laparoscopic inguinal hernia repair | * | * | * | NO | ** | NO | * | * | 7 |
| AD/EKJ/MW | 40 | van den Broeke | 2013 | Altered cortical responsiveness to pain stimuli after high frequency electrical stimulation of the skin in patients with persistent pain after inguinal hernia repair | * | * | * | NO | ** | Self report | * | * | 7 |
| AD/EKJ/MW | 39 | Moore | 2016 | Efficacy of retroperitoneal triple neurectomy for refractory neuropathic inguinodynia (Prospective study) | NO | * | * | * | ** | Self report | * | * | 7 |
| AD/EKJ/MW | 28 | Bjurström | 2017 | Neurophysiological and clinical effects of laparoscopic retroperitoneal triple neurectomy in patients with refractory postherniorrhaphy neuropathic inguinodynia | NO | * | * | NO | ** | Self report | * | * | 6 |
| AD/EKJ/MW | 13 | Bjurström | 2017 | Quantitative validation of sensory mapping in persistent postherniorrhaphy inguinal pain patients undergoing triple neurectomy | NO | * | * | NO | ** | Self report | * | * | 6 |
| AD/EKJ/MW | 7 | Kjær-Jensen | 2019 | A national center for persistent severe pain after groin hernia repair: Five-year prospective data | * | * | * | NO | ** | Self report | * | * | 7 |
| AD/EKJ/MW | 31 | Wheeler | 2019 | Evaluation of postsurgical hyperalgesia and sensitization after open inguinal hernia repair: A useful model for neuropathic pain? | * | * | * | * | ** | Self report | * | * | 8 |
| AD/EKJ/MW | 10 | Kjær-Jensen | 2020 | Trajectories in severe persistent pain after groin hernia repair: a retrospective analysis | * | * | * | NO | ** | Self report | * | * | 7 |

1. Wells GA, Wells G, Shea B, Shea B, O'Connell D, Peterson J, et al., editors. The Newcastle-Ottawa Scale (NOS) for Assessing the Quality of Nonrandomised Studies in Meta-Analyses. https://www.ohri.ca/programs/clinical_epidemiology/oxford.Asp (accessed 01/03/2023)
