# Supplementary material for: Quantitative somatosensory assessments in patients with persistent pain following groin hernia repair: A systematic review with a meta-analytical approach
Source: PLoS One. 2024 Jan 31;19(1):e0292800. doi: 10.1371/journal.pone.0292800 (PMC10830060; doi:10.1371/journal.pone.0292800)
Supplement: Attachment — Submitted filename: Response_to_reviewers_R2.docx [file pone.0292800.s006.docx]

**PONE-D-23-14833R1**

**Response to PLOS ONE second revision letter**

**Quantitative somatosensory assessments in patients with persistent pain following groin hernia repair: A systematic review with a meta-analytical approach**

Dear Academic Editor Armando Almeida,

We would like to express our gratitude for the opportunity to further revise and resubmit our manuscript to PLOS ONE. We are grateful that our work is being considered for publication in your journal.

We appreciate the careful review and constructive feedback provided by the reviewers. Your insights have been invaluable in improving the quality and clarity of our study.

Please, find below our replies to the Reviewers' comments. The Reviewers' comments are numbered chronologically (C1, C2, C3…) and according to the Reviewer’s # (R1, R2) followed by the authors' answers (A1, A2...). Page and line numbers refer to the version with track changes.

Best regards on behalf of the authors,

Akhmedkhan Dubayev, M.D., and Mads U. Werner, M.D., Ph.D.

**REVIEWER #1**

**C1R1:** *Page 8, lines 88-92: Despite the changes made by the authors, I still struggle to understand how this work will result in “enhanced prediction of prognostic outcomes”. By prognostic outcomes do you mean the QST? I would suggest to delete this sentence.*

**A1R1:** Thank you for the suggestion. We have deleted the sentence.

**C2R1:** *Page 10, lines 211-213: Beware that this sentence is repeated below (in red).*

**A2R1:** Thank you for noticing the error. We have now deleted one of the sentences.

**C3R1:** *Page 10, line 218: Thank you for this clarification. I would just advise to clarify if it is “and” or “or”, and eliminate the “and/or” option. I say this because if you write “at least one standard mechanical AND one thermal”, it means that both had to be present in the paper. On the other hand, if you write “at least one standard mechanical OR one thermal”, it means that the study would be included if only one QST modality was present. Since the sentences have very different meanings, “and/or” is not adequate. Please, select one.*

*Eligibility criteria: Please, also mention in this section that there were no restrictions in terms of age. Also, it should be clearly mentioned here that the included studies examined the respective cohorts after a post-surgical time period of at least three months.*

**A3R1:** We thank you for the comment. We agree, and we have changed it to “or” clarifying our standpoint.

**C4R1:** *Page 14, line 309: I think that the analyses could be better understood if the authors mentioned here the calculations performed (back-transformed mean differences). This would clarify which values were included in the forest plots.*

**A4R1:** Transformed values are indicated in the tabulated part of the forest plots. MDT and MPT values are not back-transformed in the forest plots. Back transformations are solely provided in the main text (page 30, line 469 and page 40, line 496).

**C5R1:** *Page 30, line 319: The authors could write here “decrease in threshold from the affected side to control side”.*

**A5R1:** The sentence has been rephrased to accommodate the suggestion.

**C6R1:** *Page 30, lines 463-464: If the information in brackets are the CI, this should be in the brackets (95% CI=1.6, 6.9)*

**A6R1:** “95% CI” has now been added in the brackets in our Results’ section.

**C7R1:** *Page 46, line 637: The second aim is very clearly stated here. I suggest writing it the same way in the abstract and aim sections.*

**A7R1:** We thank you for the comment. We have rephrased the second aim in the Abstract and the Aims (1.1.3.) section (page 4, lines 76-77 and page 8, line 168-169).

**C8R1:** *Page 47, line 664: I suggest presenting all the discussion for RoB under the subtitle 4.2. Otherwise, it is strange why the Cochrane Tool is not mentioned.*

**A8R1:** The discussion for RoB 2.0 has now been reinstated under section 4.2.

**C9R1:** *Page 54, line 836: I suggest adding some comment about the possible limitation of making comparisons with the contralateral site in some studies, but with healthy subjects in other studies.*

**A9R1:** We are grateful for the suggestion. We have written the following paragraph to comment on the issue in subsection 4.6.1. Studies (page 55, lines 842-854):

*“Healthy controls vs. contralateral side:* The controlled studies used different methodological approaches when comparing the sensory abnormalities at the surgical site to a control site. Either an *absolute* approach, comparing with a normative healthy cohort, or a *relative* approach, comparing with the individual’s contralateral homotopic site, or a combination of these approaches, were used. One of the advantages of the *relative* approach is that the within-subject variances often are significantly smaller than the between-subject variances. Using the individual’s contralateral site as a control is thus expected to reduce data variability, making the data more robust and less susceptible to confounding factors such as age, gender, and random errors. On the other hand, mirror-image sensory dysfunction [**1**], a neural cross-talk between the sides, may influence the side-to-side difference in the relative approach. Very few studies have systematically examined the pros and cons of the absolute and relative approaches [**2**].”

References

1.	Enax-Krumova E, Attal N, Bouhassira D, Freynhagen R, Gierthmühlen J, Hansson P, et al. Contralateral Sensory and Pain Perception Changes in Patients With Unilateral Neuropathy. Neurology. 2021;97(4):e389-e402.

2.	Kemler M, Schouten H, Gracely R. Diagnosing Sensory Abnormalities with Either Normal Values or Values from Contralateral Skin. Anesthesiology. 2000;93:718-27.
